# Dynamic contrast enhanced-MRI and diffusion-weighted image as predictors of lymphovascular invasion in node-negative invasive breast cancer

**DOI:** 10.1186/s12957-021-02189-3

**Published:** 2021-03-15

**Authors:** Bo Bae Choi

**Affiliations:** Department of Radiology, Chungnam National University Hospital, 282 Munhwa-ro, Jung-gu, Daejeon, 35015 Republic of Korea

**Keywords:** Breast cancer, Magnetic resonance imaging, Diffusion-weighted imaging, Lymphovascular invasion

## Abstract

**Background:**

Lymphovascular invasion (LVI) is an important risk factor for prognosis of breast cancer and an unfavorable prognostic factor in node-negative invasive breast cancer patients. The purpose of this study was to evaluate the association between LVI and pre-operative features of dynamic contrast-enhanced magnetic resonance imaging (DCE-MRI) and diffusion-weighted imaging (DWI) in node-negative invasive breast cancer.

**Methods:**

Data were collected retrospectively from 132 cases who had undergone pre-operative MRI and had invasive breast carcinoma confirmed on the last surgical pathology report. MRI and DWI data were analyzed for the size of tumor, mass shape, margin, internal enhancement pattern, kinetic enhancement curve, high intratumoral T2-weighted signal intensity, peritumoral edema, DWI rim sign, and apparent diffusion coefficient (ADC) values. We calculated the relationship between presence of LVI and various prognostic factors and MRI features.

**Results:**

Pathologic tumor size, mass margin, internal enhancement pattern, kinetic enhancement curve, DWI rim sign, and the difference between maximum and minimum ADC were significantly correlated with LVI (*p* < 0.05).

**Conclusions:**

We suggest that DCE-MRI with DWI would assist in predicting LVI status in node-negative invasive breast cancer patients.

## Background

Prognostic factors are important for the management and treatment of breast cancer. Classical prognostic factors are axillary lymph node (ALN) status, tumor size, and nuclear and histological grade, while estrogen receptor (ER), progesterone receptor (PR), and human epidermal growth factor 2 (HER2) are known as molecular prognostic factors [1, 2]. Lymphovascular invasion (LVI) is defined as the presence of tumor cells within a definite endothelial-lined space (lymphatic or blood vascular system) in the breast surrounding invasive carcinoma [3]. The presence of LVI is associated with an increased risk of ALN and distant metastases [4], and is an unfavorable prognostic indicator for breast cancer survival and recurrence [5–7]. In particular, in patients with node-negative invasive breast cancer LVI is an independent prognostic factor for survival, recurrence, and distant metastasis [8]. The presence of LVI is currently determined at surgery and recorded on the surgical pathology report, but if certain features related to LVI could be detected by pre-operative imaging, predictions on tumor prognosis including lymph node (LN) metastasis could be made, and choice of chemotherapy could be informed, prior to surgery.

There have been several reports on the association between LVI and magnetic resonance imaging (MRI) parameters [9–11], of them, only a few studies about MRI features with easy access of analysis at practical field. Shi et al. [10] analyzed the correlation of LVI with mass margin, diffusion-weighted imaging (DWI) signal, and time intensity curve, and Cheon et al. [11], in addition included peritumoral edema and adjacent vessel sign in their analysis. However, a few factors showed discordance between the two studies. To achieve a consensus on the MRI parameters that correlate with LVI, more studies with different approaches are needed.

DWI is an MRI technique based on the difference in random motion of water molecules between tissues, and is a very sensitive means of detecting cell density, membrane integrity, and tumor microstructure. The apparent diffusion coefficient (ADC) is a measure of the degree of water diffusion within tissue. DWI and the ADC are effective for discriminating benign and malignant tissue, improving the diagnosis of breast cancer when combined with dynamic contrast-enhanced MRI (DCE-MRI), and they are effective for response monitoring after neoadjuvant chemotherapy [12, 13]. ADC values have been associated with classical and molecular prognostic factors, as well as treatment response [14–16]. However, the results are discordant, with no standard value identified and limited reproducibility between the different machines, conditions, or protocols used. In studies of LVI and ADC, LVI has been shown to associate with either the medial, minimum, or peritumoral ADC, depending on the study design [17–19]. To improve generalizability and accuracy, we used the ADC difference value (ADC-dif), derived by subtracting the minimum ADC of the mass from its maximum. ADC-dif represents the heterogeneity of the tumor and is a parameter associated with several prognostic features of MRI [20, 21]. We also studied the association between DWI rim sign and LVI. The DWI rim sign is the presence of a high intensity signal around the rim of the mass, which was reported to correlate with tumor malignancy by Kang et al. [22].

The purpose of this study was to evaluate the association between various MRI features, including from DWI, and LVI in node-negative invasive breast cancer.

## Methods

### Patients

Data from patients who underwent pre-operative MRI and breast cancer surgery (mastectomy or lumpectomy) for invasive ductal carcinoma (IDC) that was not otherwise specified (NOS) between July 2017 and October 2018 were collected retrospectively from our institutional database. Cases of neo-adjuvant chemotherapy and node-positive on the surgical pathology report were excluded. Finally, 132 cases of IDC NOS type breast cancer reported as node-negative on the surgical pathology report were included in this study.

### MRI acquisition techniques and interpretation

MRI was performed with the patient in the prone position using a 1.5T scanner (Signa Excite, GE Healthcare, Milwaukee, WI, USA) equipped with a dedicated 8-channel surface breast coil. Images were acquired in the axial plane with the following sequences: axial, T2-weighted (T2W), fat-suppressed, fast spin-echo imaging (TR/TE, 5000/86; flip angle, 90°; field of view (FOV), 280–360 mm; acquisition matrix, 320 × 256; number of excitations (NEX), 3; slice thickness, 4.5 mm) and pre- and post-contrast, axial, T1-weighted (T1W) 3-dimensional (3D) fast spoiled gradient-recalled echo sequence with parallel volume imaging (VIBRANT, GE Healthcare, Oslo, Norway) (TR/TE, 6.6/3.2; flip angle, 10°; FOV, 280–360 mm; acquisition matrix, 360 × 360; NEX, 0.8; slice thickness, 1.1 mm). Gadodiamide (Omniscan, GE Healthcare) was administered as the contrast agent with an intravenous bolus injection (0.2 mmol per kg body weight) at a rate of 3 ml/s. Imaging was performed before the intravenous contrast agent bolus injection and four times afterwards for a period of 7.3 min. The image post-processing included the subtraction of unenhanced images from enhanced images, sagittal reformations, and 3D maximum-intensity projections using the first contrast-enhanced series. DWI was performed using spin-echo single shot echo-planar imaging with *b* values of 0 and 1000 s/mm^2^; TR/TE, 4000/61.6 ms; FOV, 280–360 mm; acquisition matrix, 90 × 128; NEX, 6; slice thickness, 4.5 mm; and gap, 0.6 mm. DWI was performed in each of three orthogonal directions along the *x*, *y*, and *z* axes. Images from each direction were combined to produce a single image at each slice location showing the diffusion-restricted area. The signal intensities of all three directions were combined (Scmb) so that Scmb = {Sx·Sy·Sz}/3, where Sx, Sy, and Sz are the signal intensity values of the three orthogonal directions. ADC values were calculated according to the following formula: ADC = [1/(b2 − b1)]ln(S1/S2), where S1 and S2 are the signal intensities in the regions of interest (ROIs) obtained from the two gradient factors b2 and b1 (b1 = 0 and b2 = 1000 s/mm^2^ for the 1.5T scanner).

**Fig. 1:**
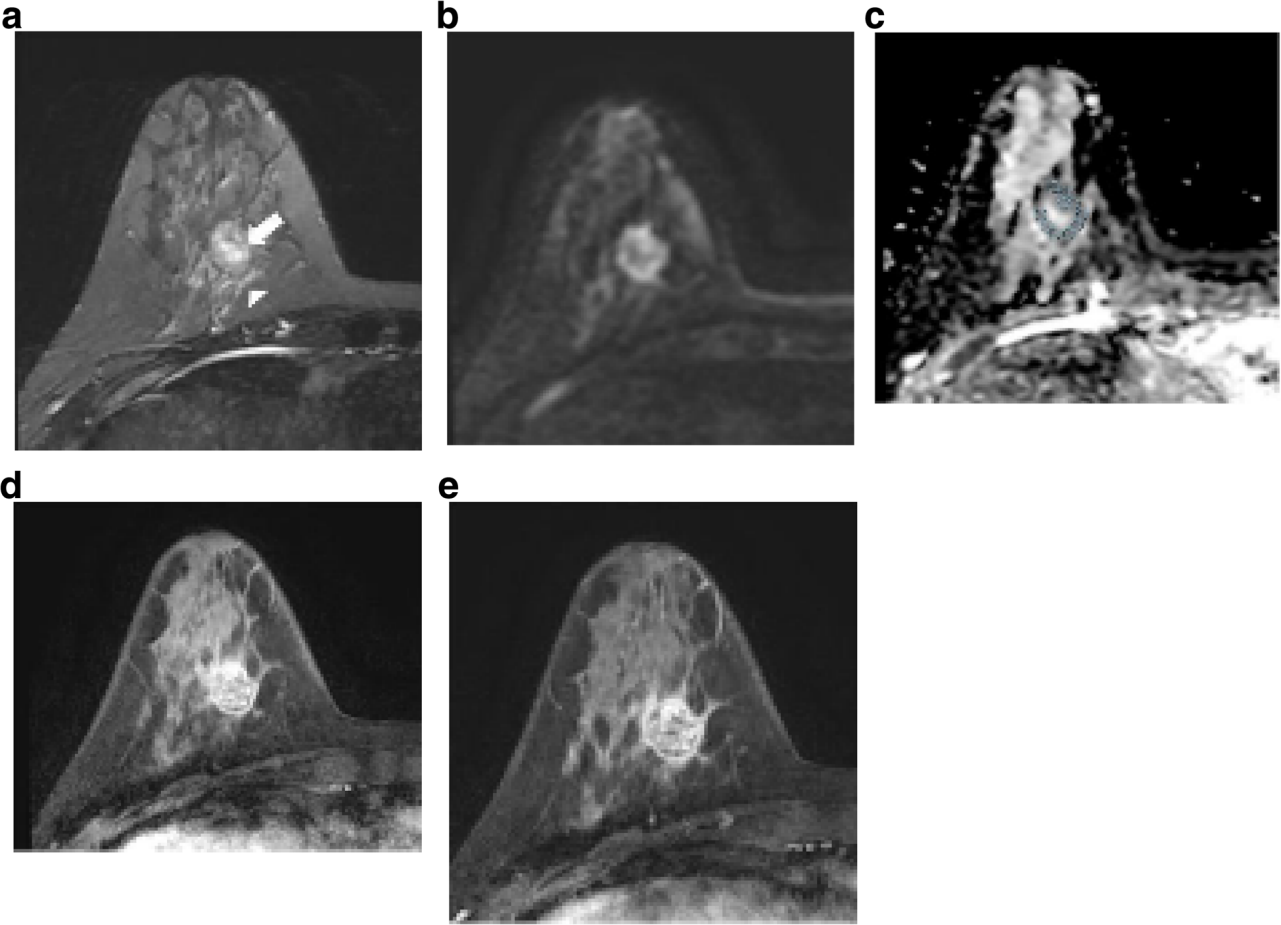
Images of a 37-year-old woman with invasive ductal carcinoma and a lymphovascular invasion. **a** Axial T2 weighted image shows a 2.3 cm mass in her right breast. Intratumoral T2 high signal (arrow) and peritumoral edema (arrowhead) are noted. **b** High signal intensity rim is shown in axial diffusion weighted image (*b* value=1000 s/mm^2^). **c** Apparent diffusion coefficient map shows restriction along the periphery of the mass. Multiple region of interests (ROI)s of 6.87 mm^2^ were manually placed within the mass avoiding cystic or necrotic area. Minimum ADC value was 1025 × 10^−6^ mm^2^/s, and maximum ADC value was 1345 × 10^−6^ mm^2^/s. The calculated ADC difference was 320 × 10^−6^ mm^2^/s. **d**, **e** Axial contrast-enhanced T1-weighted image after 2 min (**d**) and 6 min (**e**) of contrast injection demonstrating a round, circumscribed mass in right breast. This mass shows rim internal enhancement and washout kinetic pattern of enhancement. This patient underwent modified radical mastectomy of her right breast. The histopathological features of this mass were poorly differentiated, no lymph node metastasis, ER-. PR-, HER2- and Ki-67 high (70%). Tumor size at final pathologic report was 3.6 cm

The interpretation of the degree and patterns of enhancement was performed by visual assessment. One breast radiologist (with 10 years of experience) reviewed the breast cancer MRI images. The vertical and horizontal dimensions of the masses were measured on the image section showing the largest mass on early-enhancement T1W images, and the largest diameter among the vertical and horizontal dimensions was recorded. In the case of multifocal and multicentic tumors, the size was measured at the largest mass by the same method. For non-mass enhancement lesions, total extent of the lesion was measured and its size was recorded. MRI was reported using the Breast Imaging Reporting and Data System (BI-RADS) MRI lexicon [23] with respect to mass or non-mass lesion, mass shape (oval, round, irregular), mass margin (circumscribed, irregular, spiculated), and internal enhancement characteristics (homogeneous, heterogeneous, rim enhancement). Rim enhancement was defined as strong enhancement at the periphery of a tumor compared with that at the center. MRI enhancement kinetics were evaluated by visual assessment as follows: type I curve (progressive enhancement pattern), type 2 curve (plateau pattern), and type 3 curve (washout pattern). A type 2 curve was not observed in this study. Intratumoral high signal intensity on T2W images was assessed compared with surrounding normal breast parenchymal signal intensity on fat-suppressed T2W images, and was defined as very high signal (when the signal was similar to a water or vessel signal), high signal (when the mass signal was higher than that of surrounding breast tissue), and low signal (when the mass signal was lower than that of surrounding breast tissue). Peritumoral edema was assessed by visual assessment of fat-suppressed T2W images. Peritumoral edema was recorded as present when a high signal was noted posterior to the tumor mass in the pre-pectoral area, or when there was a fairly extensive high signal around the tumor mass. The ADC of a mass was measured by manually placing ROIs within a mass on the ADC maps automatically calculated by the MRI software. Axial ADC maps representing the largest diameter of the mass were selected for ROI placements. Multiple ROIs of 17–23 mm^2^ were placed within the mass, with as many as possible being examined [24] (Fig. 1c). ROIs were carefully placed to avoid cystic areas, necrotic areas, and visual DWI artifacts. The lowest ADC value from the multiple ROIs was regarded as the minimum ADC, and the highest ADC value as the maximum ADC. The difference between the maximum and minimum ADC was recorded as ADC-dif. A high signal rim surrounding a mass was recorded as a positive DWI rim sign, irrespective of whether it was complete or incomplete [22].

### Histologic analysis

Pathological reports from core needle, excisional biopsies, breast-conserving surgery, or mastectomy specimen were reviewed by two breast pathologists (with 20 and 5 years of experience). Histological analysis was carried out on specimens obtained at the last surgery. Pathologists reported tumor histologic type, invasive tumor size, and LN status. LVI was assessed on hematoxylin and eosin-stained sections, and was defined as carcinoma cells in a definite endothelial-lined space in the peritumoral breast surrounding the invasive carcinoma. Immunohistochemical staining was used to determine the expression of the following molecular markers: ER, PR, HER2, and Ki-67. ER and PR expression were quantified using the Allred scoring system, and were considered as positive for ER or PR with a total Allred score >2 [25]. The intensity of HER2 staining was scored as 0, 1+, 2+, or 3+. Tumors with a score of 3+ were classified as HER2 positive, while tumors with a score of 0 or 1+ were negative. A HER2 value of 2 was considered equivocal. For equivocal result, further testing of fluorescence in situ hybridization (FISH) was conducted, and pathologists reported the final molecular types of breast cancer. Ki-67 expression was recorded as the percentage of tumor epithelial cells determined by immunohistochemical assay, both digital image analysis and visual scoring by light microscope were used for quantification.

### Statistical analysis

Analysis of variance was performed for tumor histologic grade, expression of Ki-67, hormone receptor status, existence of LVI, and other MRI findings. For the examination of statistical differences in the clinicopathological variables and MRI features among the LVI positive (LVI+) group and LVI negative (LVI−) group, the chi-square test or Fisher’s exact test was used, and the Kruskal–Wallis test was used for histologic grade. The odds ratio (OR) and 95% confidence interval (CI) for LVI were calculated with univariate logistic regression analysis. The Kolmogorov–Smirnov test was used to test for normality in the analysis of numerical data. The numerical data except for ADC-dif were verified as being normally distributed and were analyzed using the independent sample *t* test. For ADC-dif, the Mann–Whitney *U* test was used. A *p* value of <0.05 was considered statistically significant. Statistical analyses were performed using PASW Statistics 18.0 (IBM, Armonk, NY, USA).

## Results

### Patients

The mean age of 132 cases was 54 years (range, 21-82 years). Mean size of pathologic invasive cancer was 1.8 cm (range, 0.1-5.2 cm). The most common molecular subtype was ER+ (93/132, 70.5%), and moderate differentiated was the most common histologic grade (48/132, 36.4%).

### LVI vs. clinicopathological features

Among 132 cases of IDC NOS, 42 were LVI+ and 90 were LVI− (mean age 53 vs. 54 years, respectively). Analysis of the relationship between LVI and clinicopathologic prognostic factors revealed a significant correlation between invasive tumor size (both as measured by surgical specimen and MRI) and LVI (Table 1). Histologic grade, Ki-67, and hormone receptor status showed no correlation with LVI.

**Table 1:** Clinicopathological characteristics of patients according to lymphovascular invasion status

Variables	LVI+ (*n*=42)	LVI− (*n*=90)	OR	*P* value
Mean age (years)^a^	53±1.75	54±1.20		0.494
Histologic grade			0.7 (0.4-1.1)	0.080^b^
Well	7 (16.7)	32 (35.6)		
Moderate	19 (45.2)	29 (32.2)		
Poor	16 (38.1)	29 (32.2)		
Ki-67 status			1.0 (0.5-2.1)	0.986
High (≥14%)	22 (52.4)	47 (52.2)		
Low (<14%)	20 (47.6)	42 (47.8)		
Tumor subtype			0.9 (0.6-1.5)	0.873
ER-positive	29 (69)	64 (71.1)		
HER2-positive	5 (11.9)	12 (13.3)		
Triple-negative	8 (19)	14 (15.6)		
Pathologic tumor size (cm)	2.1±0.13	1.6±0.08	0.5 (0.3-0.7)	0.001^c^
MRI tumor size	2.0±0.15	1.7±0.11	0.8 (0.5-1.1)	0.007

*ER* Estrogen receptor, *HER2* Human epithermal growth factor receptor 2

*Data are numbers of patients, with percentages in parentheses unless otherwise indicated

^a^Data are mean age of patients

^b^Kruskal-Wallis test

^c^Mann-Whitney *U* test

### LVI vs. MRI features

**Fig. 2:**
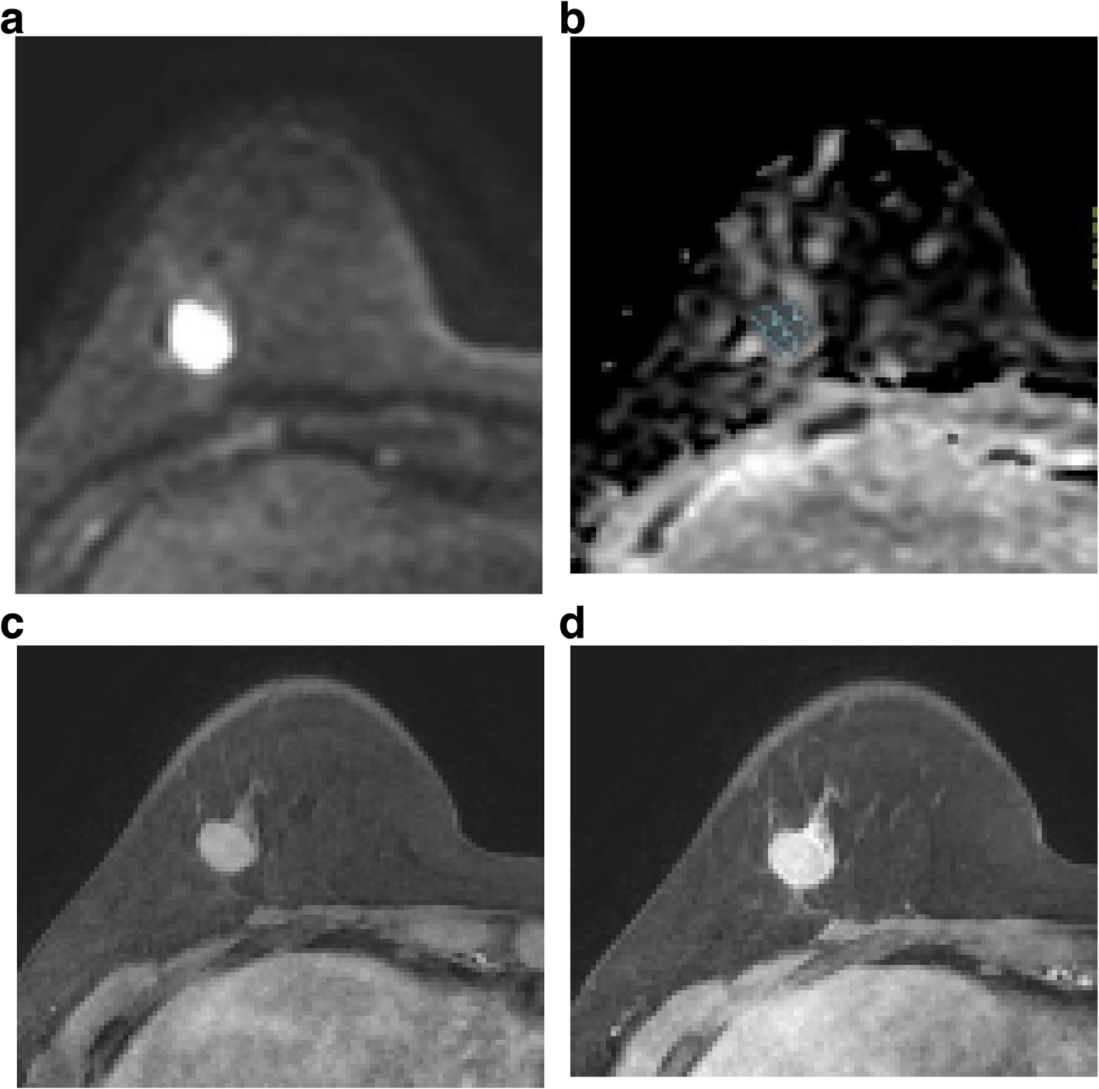
Images of a 59-year-old woman with invasive ductal carcinoma without lymphovascular invasion. **a** Diffusion-weighted image demonstrates homogeneous high signal intensity of mass without rim sign (*b* value=1000 s/mm^2^). **b** Apparent diffusion coefficient map. Multiple region of interests (ROI)s of 7.81 mm^2^ were manually placed within the mass avoiding cystic or necrotic area. Minimum ADC value was 826 × 10^−6^ mm^2^/s, and maximum ADC value was 1069 × 10^−6^ mm^2^/s. The calculated ADC difference was 243 × 10^−6^ mm^2^/s. **c**, **d** Axial contrast-enhanced T1-weighted image after 2 min (**c**) and 6 min (**d**) of contrast injection demonstrating an oval, circumscribed mass with homogeneous enhancement in her right breast. Kinetic analysis of enhancement pattern was washout pattern. This patient underwent modified radical mastectomy. The histopathological features of this mass were moderate differentiated, no lymph node metastasis, ER-, PR-, HER2+, and Ki-67 high (20%). Tumor size at final pathologic report was 1.6 cm

We found significant associations between LVI and mass margin, internal enhancement pattern, and outcome of the kinetic enhancement curve analysis (Table 2). For the LVI+ group, circumscribed, irregular, or spiculated margins occurred to similar degrees, while a circumscribed margin was the most common in the LVI− group (Figs. 1 and 2). A rim enhancement pattern of the mass was most common in the LVI+ group (Fig. 1d), while in the LVI− group it was the homogeneous pattern (Fig. 2c). In the enhancement curve analysis, a washout pattern was most common in both the LVI groups (Figs. 1 and 2), but the washout pattern showed more in the LVI+ group (85.7% vs. 66.7%). A high intratumoral T2W signal and peritumoral edema were equally uncommon in both the LVI groups (Fig. 1a), and there was no correlation with neither factor nor LVI.

**Table 2:** MRI features and lymphovascular invasion status in node-negative breast cancer patients

Variables	LVI+ (*n*=42)	LVI− (*n*=90)	OR	*P* value
Mass shape			0.6 (0.3-1.3)	0.169
Oval or round	17 (40.5)	48 (53.3)		
Irregular	25 (59.5)	42 (46.7)		
Mass margin			0.6 (0.3-0.9)	0.045
Circumscribed	15 (35.7)	46 (51.1)		
Irregular	14 (33.3)	32 (35.6)		
Spiculated	13 (31.0)	12 (13.3)		
Mass internal enhancement			0.5 (0.3-0.8)	0.007
Homogeneous	6 (14.3)	37 (41.1)		
Heterogeneous	16 (38.1)	28 (31.1)		
Rim	20 (47.6)	25 (27.8)		
Kinetic analysis			0.3 (0.1-0.9)	0.022
Progressive pattern	6 (14.3)	30 (33.3)		
Washout pattern	36 (85.7)	60 (66.7)		
Intratumoral T2 high signal			2.8 (0.9-8.3)	0.077^a^
Positive	8 (19.0)	7 (7.8)		
Negative	34 (81.0)	83 (92.2)		
Peritumoral edema			1.6 (0.6-3.8)	0.329
Positive	10 (23.8)	15 (16.7)		
Negative	32 (76.2)	76 (83.3)		
DWI rim sign			2.5 (1.2-5.4)	0.017
Positive	28 (66.7)	40 (44.4)		
Negative	14 (33.3)	50 (55.6)		

*DWI* Diffusion weighted image

*Data are numbers of patients, with percentages in parentheses unless otherwise indicated

^a^Fisher’s exact test

Analysis of the relationship between LVI and DWI findings revealed that the LVI+ group had a significantly higher incidence of the DWI rim sign than the LVI− group (Fig. 1b). Of the different types of ADC values analyzed vs. LVI, only ADC-dif was significantly correlated with LVI (*p* = 0.000, Figs. 1 and 2). The maximum ADC value was higher in the LVI+ group, but was not significantly different from than in the LVI− group (*p* = 0.059; Tables 2 and 3).

**Table 3:** Quantitative ADC parameters according to lymphovascular invasion status

Variable	LVI+ (*n*=42)	LVI− (*n*=90)	*P* value
Minimum ADC (×10^−3^ mm^2^/s)	0.917±0.023	0.931±0.020	0.674
Maximum ADC (×10^−3^ mm^2^/s)	1.163±0.216	1.092±0.190	0.059
Mean ADC (×10^−3^ mm^2^/s)	1.040±0.174	1.011±0.179	0.393
ADC difference ADC (×10^−3^ mm^2^/s)	0.246±0.134	0.162±0.111	0.0

*ADC* Apparent diffusion coefficient

## Discussion

LVI is a risk factor for ALN and distant metastasis, and helps to predict survival and local or distant recurrence in node-negative invasive breast cancer patients [4–8]. LVI status is also effective for response evaluation after neo-adjuvant chemotherapy (NAC) [3]. Previous studies have reported a correlation between LVI and well-known prognostic factors of breast cancer, including hormone receptor expression status, tumor size, ALN status, age, and histological grade [14, 26], but the results between studies are discordant [11]. In the present study, invasive tumor size was related to LVI; however, we found no correlation with histologic grade, hormone receptor status, or Ki-67 status.

LVI was correlation with morphologic and kinetic features of breast cancer on DCE-MRI. We found significant correlation between LVI and mass margin, internal enhancement pattern, and type of kinetic enhancement curve. And, this result is the novelty of our study. Shi et al. [10] reported that LVI cases showed an ill-defined margin and lobulation. Although those specific terms are not included in the BI-RADS categories, ill-defined margin and lobulation can be considered as equating to the BI-RADS terminology “not-circumscribed margin.” In our study, the LVI+ cases had a higher incidence of an irregular and spiculated margin than the LVI−, which concurs with the findings of Shi et al. [10]. Cancer induces a desmoplastic reaction in adjacent tissue with growing, and irregular or spiculated margins are noted on imaging [27, 28]. In rapidly growing aggressive cancers such as triple negative breast cancer (TNBC), the desmoplastic reaction cannot catch up with the tumor growing, resulting in a circumscribed margin visible on imaging [29]. However, the mass margin alone is not sufficient to evaluate the aggressiveness of a tumor.

Washout pattern was the most common type of kinetic enhancement curve in both LVI+ and LVI− cases, with more percentages on LVI+ (85.7% in LVI+ vs. 66.7% in LVI−) (*p* = 0.022). This result correlates with those of Shi et al. [10], showing a significant association between the presence of LVI and a plateau or washout pattern. We found an association between the presence of LVI and a heterogeneous or rim enhancement pattern (85.7%, *p* = 0.007), while Cheon et al. [11] reported no correlation between the internal enhancement pattern and LVI. A washout kinetic curve pattern or rim enhancement pattern in breast cancer are reported as prognostic factors, and are associated with higher histologic grade and negative ER status, as well as with TNBC [30–33]. Washout pattern is not usually seen in benign breast masses (specificity, 90.4%) and is considered suggestive of a malignant mass [30]. The relationship between kinetic parameters and prognostic factors is controversial; however, a significant correlation has been reported between washout curve pattern and higher histologic grade, Ki-67 positivity, and negative ER status [31]. Rim enhancement is defined as enhancement more obvious at the periphery of the mass, whether it has a thin or thick rim pattern, and has been shown to correlate with histologic grade, size, and LN status [31]. Our present study showed that the significant association between the presence of LVI and MRI feature of washout curve pattern, rim enhancement, which is helpful for prediction analysis of breast cancer prognosis with DCE-MRI.

Several studies have shown that ADC values were associated with LVI, although, among multiple ADC parameters, optimal ADC parameters to match the study results varied with study design [17–19]. In studies of the relationship between ADC and LVI, Yang et al. [19] report that the minimum ADC correlates with LVI, while Karan et al. [17] state that the median ADC associates with LVI. Our study found no significant correlation for LVI with either the minimum, maximum, or mean ADC values (only a borderline correlation was found with maximum ADC (*p* = 0.059)). Besides, the way of drawing the ROI is not always the same, which makes less reproducibility. The reason for the discordance between our results and those of others is likely to be the lack of a standard value for discriminating benign from malignant tissue, and also that there is no standard method for measuring the ROI. Depending on the measuring method used, it is possible for non-invasive tissue to be included in the ROI. To overcome this problem, various measurement methods using different aspects of the ADC have been tried, for example, by applying the values for relative ADC, peritumoral ADC, and ADC-dif [20, 24]. Mori et al. [18] used peritumoral ADC and reported that it is effective in predicting LVI in node-negative breast cancer.

Breast cancer lesions are very heterogeneous, and understanding tumor heterogeneity is essential. Intratumoral heterogeneity, the presence of heterogeneous cell populations within a tumor, is an indicator for metastatic potential and treatment resistance [32, 33]. Hence, to gain more insight into tumor composition, in our study we used ADC-dif, which can provide a more nuanced reflection of the tumor’s characteristics. ADC-dif, the difference between the maximum and minimum ADC, can highlight heterogeneity in the intratumoral cellularity. ADC-dif in combination with the minimum ADC can improve the rate of concordance with histological tumor grading and raise the diagnostic performance of breast MRI [21]. In our study, ADC-dif was significantly higher in LVI+ cases (*p* = 0.0). This result implies that the LVI+ group has more intratumoral heterogeneity than the LVI− group. Therefore, in many cases, especially of pre-operative image evaluations in node-negative patients or when it is difficult to identify LN metastasis with certainty, identifying signs of intratumoral heterogeneity by ADC-dif could be a crucial indicator for further evaluation and treatment of breast cancer.

The DWI rim sign is described by Kang et al. [22] as a high signal intensity rim on DWI outlining ≥90% (complete) or ≤90% (incomplete) of the lesion. Rim sign negativity is defined as no visible high signal on the rim of the lesion on DWI. The DWI rim sign on breast lesions is associated with malignancy [22, 34], but there have been no studies on the influence of the DWI rim sign on prognosis. Choi et al. [34] hypothesized that as DWI rim sign reflects higher cellularity, its presence would vary with breast cancer subtypes, but they found no significant difference in DWI rim sign between two breast cancer subtypes. In our study, 66.7% of LVI+ cases showed the DWI rim sign compared with 44.4% of LVI− cases, suggesting that DWI rim sign is an important prognostic indicator. We, therefore, analyzed correlations between DWI rim sign and histologic grade, Ki-67, pathologic invasive tumor size, and tumor subtype. The DWI rim sign was significantly correlated with histologic grade, pathologic invasive tumor size, and tumor subtype (ER+ or ER−), and showed borderline correlation with Ki-67 (Table 4). Although we studied only node-negative breast cancer, the DWI rim sign would have a strong relationship with prognostic factors of any cancer.

**Table 4:** Clinicopathologic prognostic factors according to DWI rim sign

Variables	DWI rim sign + (*n*=68)	DWI rim sign − (*n*=64)	*P* value
Histologic grade			0.005^a^
Well	14 (20.6)	25 (39.1)	
Moderate	24 (35.3)	24 (37.5)	
Poor	30 (44.1)	15 (23.4)	
Ki-67 status			0.057
High (≥14%)	41 (60.3)	28 (43.8)	
Low (<14%)	27 (39.7)	36 (56.3)	
Tumor subtype			0.054
ER-positive	42 (61.8)	51 (79.7)	
HER2-positive	10 (14.7)	7 (10.9)	
Triple-negative	16 (23.5)	6 (9.4)	
Tumor subtype			0.024
ER-positive	42 (61.8)	51 (79.7)	
ER-negative	26 (38.2)	13 (20.3)	
Pathologic tumor size (cm)	2.1±0.10	1.4±0.89	0.0

*DWI* Diffusion weighted image, *ER* Estrogen receptor, *HER2* Human epithermal growth factor receptor 2

*Data are numbers of patients, with percentages in parentheses unless otherwise indicated

^a^Kruskal-Wallis test

Peritumoral edema and high intratumoral T2W signal on MRI are prognostic factors associated with worse recurrence-free survival [35, 36]. A few studies have demonstrated the association between LVI and peritumoral edema in breast cancer [11, 37]. Peritumoral edema is due to increased vascular permeability and peritumoral cytokines [38], and is a biomarker of aggressive breast cancer, positively associated with larger tumor size, high histologic grade, high Ki-67 value, more metastatic LNs, and recurrence [39, 40]. In the present study, we found no correlation between LVI and peritumoral edema. Similarly, Mori et al. [18] reported no association between LVI and peritumoral edema, but they did find a correlation between peritumoral ADC and LVI. To measure peritumoral ADC value, ROIs were placed where the ADC values visually appeared to be most increased adjacent to the tumor border [18]. Aggressive breast cancers have higher peritumoral ADC values, reflecting peritumoral edema and increased cell density caused by proliferative changes [41]. Thus, peritumoral ADC might be a more sensitive means of detecting early edema than signs seen on DCE-MRI. High intratumoral T2W signal is not common finding, but is associated with TNBC (25-48%) [42]. This finding correlates with a higher maximum standard unit value on positron emission tomography-computed tomography [43]. In our study, there was no correlation between LVI and intratumoral T2 high signal. Since this study is only for LN negative patients, we will get better results with larger study encompassing a range of all LN+ and LN− patients.

This study has several limitations. First, its retrospective nature and data review by only one radiologist at a single institution. The results would be more convincing if the MRI data could be reviewed by more than one radiologist. Second, drawing ROIs on very small tumors (<0.5 cm) and making multiple ROI measurements of them was difficult. The difficulty in drawing very small ROIs on images retrieved from the picture archiving and communication (PACS) system is a technical drawback of the system; thus, more molecular or biological assessment is needed for very small cancers. Third, this study targeted node-negative invasive breast cancer. Certain factors cannot be generalized to our result and need more generalized data for more accurate outcomes. Forth, it is known that the diagnostic performance of the kinetic analysis of NME in MRI is not as good as that of the mass [44–46]. In this study, there were only 4 cases of NME, so comparative analysis of NME and mass was not performed. The reason there are few NME cases is probably because IDC is the only target. If a study including all DCIS, ILC, and other types of breast cancer is conducted in the future, and NME cases are sufficiently included, a detailed analysis will be possible.

## Conclusions

We found that in node-negative invasive breast cancer, LVI was associated with various clinicopathologic prognostic factors and MRI features. DWI with DCE-MRI is helpful in predicting LVI.

## Data Availability

Data of this article is collected by our institutional own research program, which is limited to our institution.
